# Outcomes of treatment with CHOP and EPOCH in patients with HIV associated NHL in a low resource setting

**DOI:** 10.1186/s12885-020-07305-2

**Published:** 2020-08-24

**Authors:** Clement D. Okello, Abrahams Omoding, Henry Ddungu, Yusuf Mulumba, Jackson Orem

**Affiliations:** Uganda Cancer Institute, Upper Mulago Hill Road, P.O. Box 3935, Kampala, Uganda

**Keywords:** Treatment outcome, DA-EPOCH CHOP, HIV associated NHL, Low resource settings

## Abstract

**Background:**

The optimal chemotherapy regimen for treating HIV associated NHL in low resource settings is unknown. We conducted a retrospective study to describe survival rates, treatment response rates and adverse events in patients with HIV associated NHL treated with CHOP and dose adjusted-EPOCH regimens at the Uganda Cancer Institute.

**Methods:**

A retrospective study of patients diagnosed with HIV and lymphoma and treated at the Uganda Cancer Institute from 2016 to 2018 was done.

**Results:**

One hundred eight patients treated with CHOP and 12 patients treated with DA-EPOCH were analysed. Patients completing 6 or more cycles of chemotherapy were 51 (47%) in the CHOP group and 8 (67%) in the DA-EPOCH group. One year overall survival (OS) rate in patients treated with CHOP was 54.5% (95% CI, 42.8–64.8) and 80.2% (95% CI, 40.3–94.8) in those treated with DA-EPOCH. Factors associated with favourable survival were BMI 18.5–24.9 kg/m^2^, (*p* = 0.03) and completion of 6 or more cycles of chemotherapy, (*p* < 0.001). The overall response rate was 40% in the CHOP group and 59% in the DA-EPOCH group. Severe adverse events occurred in 19 (18%) patients in the CHOP group and 3 (25%) in the DA-EPOCH group; these were neutropenia (CHOP = 13, 12%; DA-EPOCH = 2, 17%), anaemia (CHOP = 12, 12%; DA-EPOCH = 1, 8%), thrombocytopenia (CHOP = 7, 6%; DA-EPOCH = 0), sepsis (CHOP = 1), treatment related death (DA-EPOCH = 1) and hepatic encephalopathy (CHOP = 1).

**Conclusion:**

Treatment of HIV associated NHL with curative intent using CHOP and infusional DA-EPOCH is feasible in low resource settings and associated with > 50% 1 year survival.

## Background

Globally, the estimated incidence of non-Hodgkin’s lymphoma (NHL) was 5/100,000 in 2012 [1]. In Uganda, the incidence of NHL was 1426/100,000 from the year 1991–2010 [2]. In 2016, an estimated 1.4 million people in Uganda were living with HIV [3]. The incidence of NHL remains significantly higher in HIV-positive patients compared with the HIV negative patients, even in the era of combination antiretroviral therapy (ART) [4–7]. The outcomes for patients with HIV-associated NHL and non-HIV associated NHL treated with chemotherapy in resource limited settings is still disappointingly low [8]. Notwithstanding, the introduction of combination ART resulted in reduced morbidity and mortality from HIV infection [9–11], and improved NHL specific outcomes [12].

The optimal chemotherapy regimen for the treatment of HIV associated NHL in low resource settings is still unclear. First line chemotherapy regimens used to treat HIV associated NHL in the post ART era include, but not limited to the infusional cyclophosphamide–doxorubicin–etoposide (CDE) with complete remission rate (CR) of 42%, median survival time of 17.8-month, and 1-year survival rate of 55% [13]; and Cyclophosphamide, Doxorubicin, Vincristine and Prednisolone (CHOP) regimen with complete remission in 57.6 and 47% in patients treated with R-CHOP and CHOP respectively, with an overall survival of about 35 months for R-CHOP and 28 months for CHOP [14].

Dose adjusted Etoposide, Prednisone, Vincristine, Cyclophosphamide, and Doxorubicin (DA-EPOCH) infusional chemotherapy is a relatively recent combination in this category. Hitherto, it has been reported to achieve 79% CR rate and 72% overall 2-year survival rate in patients with HIV associated NHL [15].

Use of the DA-EPOCH regimen has been suggested as unreasonable in low resource settings [16] possibly due to lack of infrastructure and supportive medications in addition to the demands of the 24 h continuous infusion of this regimen. Uganda Cancer Institute (UCI) has in the recent past embarked on the use of DA-EPOCH regimen in a selected group of patients with HIV associated NHL. Due to the uncertainty regarding the optimum treatment of HIV associated NHL and the paucity of published data regarding the use of DA-EPOCH in resource limited settings, we undertook a retrospective study to describe the treatment outcomes in patients with HIV associated NHL treated with DA-EPOCH and CHOP regimens at the Uganda Cancer Institute.

## Methods

### Study setting and design

Charts of patients with a diagnosis of HIV and NHL treated with either CHOP or DA-EPOCH chemotherapy regimens at the Uganda Cancer Institute (UCI) from 2016 to 2018 were retrospectively studied. Additional therapy with rituximab is limited in Uganda due to its high cost. UCI is the only tertiary cancer treatment facility in Uganda. It receives patients from the entire country, with some patients traveling over 600 km to seek treatment. Diagnosis of NHL at the UCI is based on morphological examination of the haematoxylin and eosin (H&E) stained tissues. Patients who can afford additional immunohistochemistry undertake them from private laboratories. Staging of NHL is based on the Ann Arbor staging system [17]. There is no national medical insurance cover in Uganda. Aggressive HIV associated NHLs are treated with CHOP; however, since the year 2016, a selected group of patients have been treated with DA-EPOCH based on the physicians’ judgement.

### Study procedure

Charts of eligible patients were consecutively identified by the Records Officer. Patients treated with CHOP received cyclophosphamide 750 mg/m^2^ IV on day 1, doxorubicin 50 mg/m^2^ IV bolus on day 1, vincristine 1.4 mg/ m^2^ IV bolus (max dose 2 mg) on day 1, and prednisolone 60 mg/m^2^ orally on days 1–5, repeated every 21 days for 6–8 cycles on either outpatient or inpatient basis. Patients treated with DA-EPOCH received etoposide 50 mg/m^2^ + doxorubicin 10 mg/m^2^ IV + vincristine 0.4 mg/m^2^ infusion for 24 h on days 1–4, cyclophosphamide 750 mg/m^2^ IV on day 5, and prednisolone 60 mg/m^2^ PO on days 1–5 on an inpatient basis; the DA-EPOCH dosages were adjusted based on nadir neutrophil counts in the preceding treatment cycle. All infusions were administered through peripheral intravenous lines. Normal saline was infused concurrently with the DA-EPOCH regimen through a Y-junction pot until the chemotherapy infusion was completed. No patient received rituximab or G-CSF. Data concerning additional medications, especially ART and Pneumocystis jiroveci pneumonia (PCP) were included. Adverse events were recorded at the time of each chemotherapy cycle and graded according to the NCI Common Terminology Criteria for Adverse Events (CTCAE) v5. In participants who had CT scans at baseline and end of therapy, treatment response was assessed using the Lugano Criteria but without the use of positron Emission Tomography. The last date of hospital review or death was recorded for survival analysis. Data was manually abstracted from charts using a standard data collection tool, coded, and then double entered into a computer using Epidata version 3.1 (Epidata association, Denmark) before exporting into STATA Version 14 (StataCorp, USA) for analysis.

### Study variables

The study variables included participant’s age and sex; type and stage of lymphoma, comorbidities, baseline ECOG performance score, body mass index (BMI), and number of chemotherapy cycles received, ART regimen and other additional concomitant medications received, and B-symptoms; nadir complete blood count (CBC) post chemotherapy and other adverse events, and disease response.

### Data analysis

Continuous variables were expressed as means and standard deviation (SD) if normally distributed or medians and inter quartile ranges (IQR) if skewed; categorical variables were described using frequencies and percentages; the overall treatment response rates (complete response, partial response) were estimated for both CHOP and DA-EPOCH chemotherapy arms using the binomial proportion and its 95% confidence interval as separate categories using the total number of participants enrolled at baseline as the denominator in each study arm. The proportion of patients who completed each treatment regimen were described as separate categories using the total number of participants enrolled at baseline as the denominator in each study arm. The one year overall survival rate was described for patients in the two treatment regimens using the Kaplan-Meier curves. Cox proportional hazards model was constructed to evaluate the association between patient characteristics and OS. Hazard ratios and 95% confidence intervals were generated, with hazard ratio < 1.0 indicating survival benefit.

## Results

### Baseline clinical characteristics

Charts of 120 patients were identified (CHOP, *n* = 108; DA-EPOCH, *n* = 12). The commonest histological diagnosis in the DA-EPOCH group was DLBCL, 7(58%) while for the CHOP group was Diffuse Large Cell lymphoma (DLCL), 65(60%) (Table 1). Diagnosis of DLCL was obtained from the H&E stain with no additional IHC. All patients in the DA-EPOCH group and a majority of patients in the CHOP group (*n* = 105; 97.2%) were already receiving ART prior to initiation of chemotherapy. Only 3 patients (2.8%) in the CHOP group initiated ART after completion of their chemotherapy cycles. Ten patients (83%) in the DA-EPOCH group were receiving tenofovir/lamivudine/efavirenz, one was receiving zidovudine/lamivudine/efavirenz, and the other was receiving tenofovir/lamivudine/nevirapine. Patients in the CHOP group received a variety of ART combination with 42(39%) patients receiving either tenofovir or zidovudine in combination with lamivudine and efavirenz. The rests of the patients received other combinations of first line ART. No patient had ART interrupted while receiving chemotherapy. All patients in the DA-EPOCH group and a majority of patients in the CHOP group (*n* = 99, 92%) were receiving cotrimoxazole for PCP prophylaxis. The rest were either on dapsone, or none. Markers for HIV control were not documented as these were collected at a separate ART treatment centre, independent of the UCI.

**Table 1:** Demographic factors and baseline characteristics

Variable	All patients ***n*** = 120	CHOP, ***n*** = 108	DA-EPOCH, n = 12
**Sex, n(%)**			
Male	64 (53)	53 (49)	11(92)
Female	56 (47)	55 (51)	1 (8)
**Mean age, years (SD)**	40 (10)	40(10.2)	42(8.1)
**Mean BMI, kg/m**^**2**^ **(SD)**	22 (4.7)	21 (4.6)	24(4.4)
**NHL type, n(%)**			
DLBCL	34 (28)	27 (25)	7(58)
DLCL (IHC not done)	69 (58)	65 (60)	4(33)
PBL	5 (4)	4 (4)	1(8)
NHL Other	9 (8)	9 (8)	0
Burkitt’s	3 (3)	3 (3)	0
**ECOG score, n(%)**			
0	9 (8)	8 (7)	1(8)
1	29 (24)	22 (20)	7(58)
2	12 (10)	12 (11)	0
3	7 (6)	6 (6)	1(8)
Not assessed	63 (53)	60 (56)	3(25)
**B-Symptoms**			
Yes	65 (54)	58 (54)	7(58)
No	55 (46)	50 (46)	5(42)
**Comorbidity**			
Yes	17 (14)	14 (13)	3(25)
No	103(86)	94 (87)	9(75)
**Stage**			
I	4 (3)	4 (4)	0
II	21(18)	17(16)	4(33)
III	54 (45)	48(44)	6(50)
IV	21 (18)	19(18)	2(17)
Not assessed	20 (17)	20(19)	0

**NB:** *DLBCL* Diffuse large B-cell lymphoma, *DLCL* Diffuse large cell lymphoma, *ECOG* Easter Cooperative Oncology Group, *IHC* Immunohistochemistry, *PBL* Plasmablastic lymphoma; Comorbidity referred to the presence of any other diagnosis besides NHL

### Treatment completion

Fifty one (47%) patients in the CHOP group and 8(67%) patients in the DA-EPOCH group completed 6 or more cycles of chemotherapy. Those who completed 3–5 cycles were 31(29%) in the CHOP group and 1(8.3%) in the DA-EPOCH group. Three patients in each group received less than 3 cycles of chemotherapy. Reasons for non-completion of chemotherapy cycles were serious adverse events (*n* = 12, 10%), other reasons (*n* = 4, 3%), and were not described in 104(87%) patients. Nineteen patients (18%) in the CHOP group and 3(25%) in the DA-EPOCH group had serious adverse events detected. Most were laboratory adverse events like neutropenia (CHOP = 13, 12%; DA-EPOCH = 2, 17%), anaemia (CHOP = 12, 12%; DA-EPOCH = 1, 8%), and thrombocytopenia (CHOP = 7, 6%; DA-EPOCH = 0). Others were sepsis (CHOP = 1), treatment related death (DA-EPOCH = 1) and hepatic encephalopathy (CHOP = 1), (Table 2). The lowest neutrophil count recorded was 0.12 × 10^3/uL after the first cycle of chemotherapy in a patient treated with DA-EPOCH.

**Table 2:** Treatment Outcomes

Variable		CHOP n = 108	DA-EPOCH n = 12
**RESPONSE TO TREATMENT, n(%)**			
Complete response (CR)		29 (27)	5 (42)
Partial response (PR)		14 (13)	2 (17)
Progressive disease (PD)		15 (14)	1 (8)
No response assessment		50 (46)	4 (33)
**ADVERSE EVENTS**			
**Neutropenia, n(%)**		13 (12)	2 (17)
Grade	< 2	0	0
	3	6 (6)	2 (17)
	4	7 (6)	0 (0)
**Anaemia, n(%)**		13 (12)	1 (8)
**Grade**			
	< 2	4 (4)	1 (8)
	3	9(8)	0(0)
**Thrombocytopenia, n(%)**		7 (6)	0 (0)
**Grade**	< 2	6(3)	0(0)
	**3**	1(1)	0(0)
**Other Adverse Events, n(%)**		4 (3)	0 (0)
Sepsis		1 (1)	0 (0)
Death		0 (0)	1 (8)
Hepatic Encephalopathy		1 (1)	0 (0)

**Note:** Nadir levels of neutrophil, haemoglobin and platelet counts were recorded after each chemotherapy cycles; adverse events were classified using the NCI CTCAE v5.0

### Treatment response and survival

Overall treatment response rate was 40% in the CHOP group and 59% in the DA-EPOCH group. Complete response (CR) was achieved in 29(27%) patients in the CHOP group and 5(42%) patients in the DA-EPOCH group. Partial response was observed in 14(13%) patients in the CHOP group and 2(17%) patients in the DA-EPOCH group. (Table 3).

**Fig. 1:**
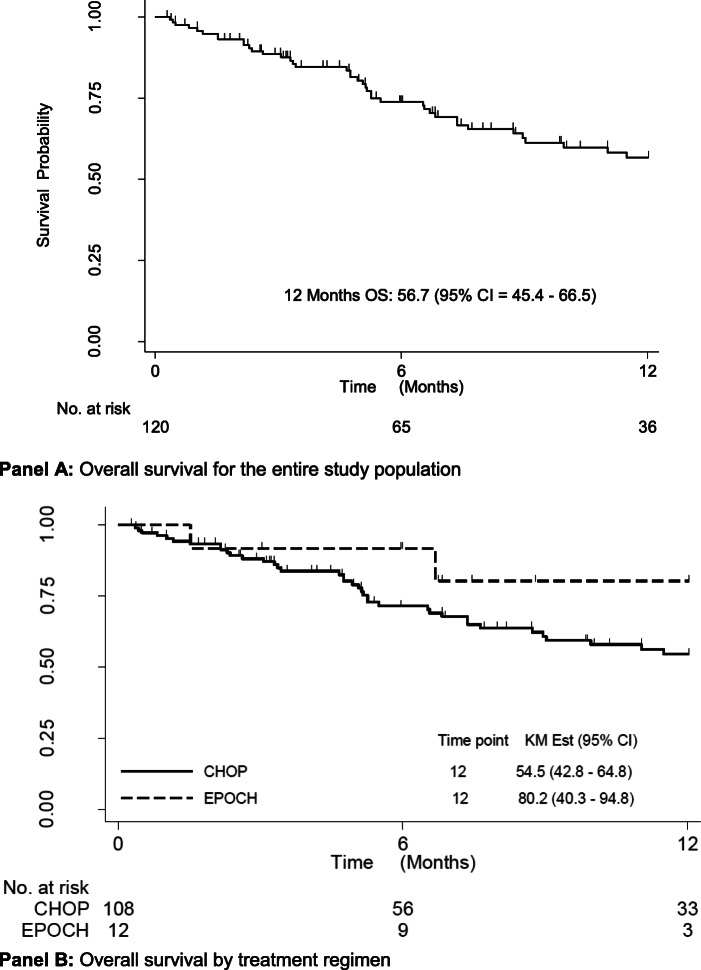
Overall survival graphs

The entire study population had a 1 year (12 months) overall survival (OS) rate of 56.7% (95% CI, 45.4–66.5), (Fig. 1, Panel A). Patients treated with CHOP had a 1 year OS of 54.5% (42.8–64.8) and those treated with DA-EPOCH of 80.2% (95% CI, 40.3–94.8), (Fig. 1, Panel B). Subset analysis for patients with DLBCL showed a 1 year OS rate of 56.1% (95% CI, 33.0–74.0) in the CHOP group and 100% in the DA-EPOCH group. Predictors of survival were analysed using patients’ age, sex, type of chemotherapy received, completion of 6 or more cycles of chemotherapy, type of lymphoma, stage of lymphoma, presence of B-symptoms and comorbidities. At univariable analysis, factors that were associated with favourable survival were ECOG performance score of 3–4, BMI 18.5–24.9 kg/m^2^ and completion of 6 or more cycles of chemotherapy. However, at multivariable analysis, only BMI 18.5–24.9 kg/m^2^ (normal BMI), (*p* = 0.03) and completion of 6 or more cycles of chemotherapy, (*p* < 0.001) were favourably associated with survival, Table 3.

## Discussion

This retrospective study highlighted that treatment of HIV-associated NHL with curative intent using CHOP and infusional DA-EPOCH is feasible in a low resource setting.

The one year OS of patients treated with CHOP and DA-EPOCH in our study is comparable to other results in Africa. A study in Malawi reported a 1 year OS of 59.4% in patients with HIV-associated lymphomas treated with CHOP [18]; in Botswana, the 1 year survival rate in patients with DLBCL was 52.8% following treatment with CHOP(+R) [19]; a retrospective study in south Africa on patients with HIV associated DLBCL treated with CHOP and concomitant ART reported a 2 year OS of 40.5% [20]. It has been noted that the outcomes of treating aggressive B cell NHL with chemotherapy appear to be similar in HIV-positive and HIV-negative populations especially in the era of combination ART [7, 21, 22]. Some studies report a CD4 count < 100/uL as a negative prognostic finding [14, 23]. However, our study did not have data on CD4 counts.

Normal BMI and completion of 6 or more cycles of chemotherapy were associated with favourable survival in our study. A retrospective study on HIV associated lymphomas in Nigeria reported stage of lymphoma as the only factor predictive of survival [24]. Other factors that have been noted to predict survival include type of lymphoma [25], age, ECOG performance scores, stage of lymphoma and LDH level [26].

We acknowledge that the observed differences in OS and response rates between the CHOP and DA-EPOCH groups in our study does not demonstrate any real differences given the different characteristics of the patients, and especially the small number of patients in the EPOCH group. However, the initial study on DA-EPOCH in patients with DLBCL reported better OS rate at 62 months of 73% than with CHOP [27]. Subsequent addition of Rituximab to DA-EPOCH produced even better results of a 12-month PFS rate of 85% [28, 29]. Additionally, a study by the AIDS-Malignancies Consortium Trial 010, a phase 3 trial of CHOP vs R-CHOP in patients with HIV-associated NHL showed a better CR of 47% for CHOP [14] than was observed in our study (27%).

Other studies on the treatment of HIV associated NHL with CHOP or DA-EPOCH in the sub-Saharan Africa show similar results with our study. De Witt (2013) in their retrospective study on patients with HIV associated DLBCL treated with CHOP (*n* = 34) and CHOP-like (*n* = 2) regimens in south Africa reported CR of 38.9% [20]. A smaller study in Malawi (*n* = 12) on patients with plasmablastic lymphoma in HIV positive (*n* = 6) and HIV negative patients (n = 6) in which 8 patients were treated with CHOP and 4 patients were treated with modified DA-EPOCH reported an overall CR in 42% of the patients (CHOP = 25%; DA-EPOCH = 75%) [30]. In another retrospective study in south Africa where only 4 cases (< 1%) were HIV(+) and no specific chemotherapy regimens were defined, the overall CR range was 46–75% for all subtypes of NHL [31]; and in a large retrospective study of paediatric Burkitt’s Lymphoma in Uganda where 70 of the 228 patients were HIV positive with a mean age of 6.7 years and no specific chemotherapy was mentioned, CR was 36% [32].

The low completion rate of chemotherapy in our study may have partly contributed to the low treatment response rates. However, adverse events contributed to non-completion of chemotherapy in only 10% of the patients whereas a majority of patients did not have clearly documented reasons for non-completion of chemotherapy.

Haematological adverse events (AEs) were the most prevalent in our study population. However, this should be taken with caution due to the limitation associated with data abstraction from patient charts that may not easily capture non lab based AEs. Takondwa et al. [30] in their study in Malawi reported treatment delays in patients receiving DA-EPOCH (*n* = 4/4) and patients treated with CHOP (n = 4/8) due to grade 3/4 neutropenia and grade 3 anaemia (CHOP = 1). It is possible that the small number of patients in our study and that by Takondwa et al. [30] may not have been sufficient to adequately evaluate the AEs. Despite the infusion of DA-EPOCH through peripheral lines, there were no other major documented concerns in the patients who received it.

To the best of our knowledge, our study is one of the few studies to describe CHOP and DA-EPOCH regimens in the treatment of HIV associated NHL in the sub-Saharan Africa. However, we acknowledge the following limitations: the imbalance between the two groups in terms of the sample size and the other baseline characteristics that limited meaningful comparisons, inadvertent patient selection bias to the treatment groups – moreover, recent treatment approaches might have favoured patients treated with DA-EPOCH, lack of HIV characteristics, limited data on chemotherapy toxicities that might have resulted from inadequate documentation by the treating physicians, and lack of immunohistochemistry to refine the diagnosis. It is possible that some patients who were treated with the CHOP regimen might have had more aggressive histological subtypes of NHL such as Burkitt lymphomas or plasmablastic lymphomas that were treated inadequately. CHOP regimen is considered less intensive and therefore inadequate for the treatment of Burkitt lymphoma [33, 34] and Plasmablastic lymphoma [35]. All these may limit the generalizability of our findings.

## Conclusion

This study showed that treatment of HIV-associated NHL with curative intent using CHOP and infusional DA-EPOCH is feasible in low resource settings and associated with > 50% one year survival. Additional studies are required to prospectively explore this observation in similar settings.

## Data Availability

All data generated or analysed during this study are included in this published article.

## Appendix

**Table 3:** Predictors Of Survival

Variable	Univariable		Multivariable	
	CHR (95%CI)	***P-***Value	AHR (95%CI)	***P-***Value
**Age in years**	1.0 (0.98–1.05)	0.32	1.0 (0.96–1.04)	0.95
**Sex: Male**	1.2 (0.64–2.24)	0.57	1.6 (0.76–3.32)	0.22
**Study arm: EPOCH**	0.4 (0.10–1.78)	0.24	0.6 (0.13–2.84)	0.52
**ECOG**				
0–2				
3–4	3.4 (1.10–8.82)	0.01	1.7 (0.55–5.26)	0.36
Not assessed	2.3 (1.01–5.05)	0.05	1.5 (0.60–3.80)	0.39
**BMI**				
< 18.5 Kg/m^2^				
18.5–24.9 kg/m^2^	0.4 (0.20–0.83)	0.01	0.4 (0.18–0.89)	0.03
> 25 kg/m^2^	0.5 (0.23–1.33)	0.19	0.6 (0.21–2.02)	0.45
**Lymphoma stage**				
Early stage				
Late stage	1.8 (0.76–4.04)	0.19	1.6 (0.58–4.60)	0.35
Not assessed	1.5 (0.49–4.32)	0.50	1 (0.28–3.94)	0.95
**Type of lymphoma**				
Diffuse large B-cell lymphoma				
Diffuse Large cell lymphoma	1.2 (0.58–2.63)	0.58	1.0 (0.39–2.43)	0.95
Plasmablastic lymphoma	2.1 (0.58–7.75)	0.25	1.4 (0.30–6.02)	0.69
NHL Other	2.5 (0.84–7.28)	0.10	1.7 (0.52–5.74)	0.37
Burkitt’s	1.0 (0.12–7.45)	0.96	0.3 (0.03–3.45)	0.33
**Presence of B-symptoms**	0.9 (0.47–1.63)	0.67	0.9 (0.44–1.96)	0.85
**Presence of comorbidity**	0.7 (0.24–1.91)	0.46	0.9 (0.28–2.69)	0.81
**Chemotherapy cycles received**				
< 6				
> 6	0.2 (0.11–0.43)	< 0.001	0.2 (0.10–0.47)	< 0.001

Note: *AHR* Adjusted Hazard Ratio, *BMI* Body Mass Index, *CHR* Crude Hazard Ratio, *ECOG* Eastern Cooperative Oncology Group

## Abbreviations

ART: Antiretroviral Therapy
CHOP: Cyclophosphamide, Doxorubicin, Vincristine and Prednisolone
CTEP-AERS: Cancer Therapy Evaluation Program Adverse Event Reporting System
EPOCH: Etoposide, Doxorubicin, Vincristine, Cyclophosphamide, and Prednisone
HIV: Human Immunodeficiency Virus
UCI: Uganda Cancer Institute
